# Recombinant actin-depolymerizing factor of the apicomplexan *Neospora caninum* (NcADF) is susceptible to oxidation

**DOI:** 10.3389/fcimb.2022.952720

**Published:** 2022-12-19

**Authors:** Luciana Baroni, Péricles Gama Abreu-Filho, Luiz Miguel Pereira, Markus Nagl, Ana Patricia Yatsuda

**Affiliations:** ^1^ Faculdade de Ciências Farmacêuticas de Ribeirão Preto, Universidade de São Paulo, Ribeirão Preto, Brazil; ^2^ Institute of Hygiene and Medical Microbiology, Medical University of Innsbruck, Innsbruck, Austria

**Keywords:** actin-depolymerizing factor (ADF), *Neospora caninum*, Apicomplexa, actin-binding protein, redox, N-chlorotaurine, taurine chloramine

## Abstract

*Neospora caninum* is a member of Apicomplexa Phylum and the causative agent of neosporosis, a disease responsible for abortions in cattle. Apicomplexan parasites have a limited set of actin-binding proteins conducting the regulation of the dynamics of nonconventional actin. The parasite actin-based motility is implicated in the parasite invasion process in the host cell. Once no commercial strategy for the neosporosis control is available, the interference in the parasite actin function may result in novel drug targets. Actin-depolymerization factor (ADF) is a member of the ADF/cofilin family, primarily known for its function in actin severing and depolymerization. ADF/cofilins are versatile proteins modulated by different mechanisms, including reduction and oxidation. In apicomplexan parasites, the mechanisms involved in the modulation of ADF function are barely explored and the effects of oxidation in the protein are unknown so far. In this study, we used the oxidants N-chlorotaurine (NCT) and H_2_O_2_ to investigate the susceptibility of the recombinant *N. caninum* ADF (NcADF) to oxidation. After exposing the protein to either NCT or H_2_O_2_, the dimerization status and cysteine residue oxidation were determined. Also, the interference of NcADF oxidation in the interaction with actin was assessed. The treatment of the recombinant protein with oxidants reversibly induced the production of dimers, indicating that disulfide bonds between NcADF cysteine residues were formed. In addition, the exposure of NcADF to NCT resulted in more efficient oxidation of the cysteine residues compared to H_2_O_2_. Finally, the oxidation of NcADF by NCT reduced the ability of actin-binding and altered the function of NcADF in actin polymerization. Altogether, our results clearly show that recombinant NcADF is sensitive to redox conditions, indicating that the function of this protein in cellular processes involving actin dynamics may be modulated by oxidation.

## Introduction

*Neospora caninum*, an obligate intracellular parasite, is successfully transmitted by congenital route in bovine cattle and may cause clinically important reproductive issues in cattle and dogs (Dubey and Schares, 2011; Dubey et al., 2017). Neosporosis, the disease caused by *N. caninum*, is considered one of the primary causes of infectious abortion in cattle, assuming considerable economic losses to the cattle industry, estimated at more than 1 billion dollars annually worldwide (Reichel et al., 2013). *N. caninum* belongs to the Apicomplexa Phylum, which parasites are known to have a nonconventional actin and few actin-binding-proteins (ABPs) compared to higher eukaryotes (Baroni et al., 2019; Frénal et al., 2020; Das et al., 2021).

Actin-depolymerizing factor and cofilin (ADF/cofilin) is a ubiquitous family of proteins found in eukaryotes and primarily described as ABP (Maciver and Hussey, 2002). More recently, the characterization of functions in other cellular mechanisms positioned these proteins as multifaceted cellular players (Kanellos and Frame, 2016). The role of ADF/cofilins in actin dynamics regulation has been investigated in many organisms (Maciver and Hussey, 2002; Poukkula et al., 2011), including in apicomplexan parasites (Schüler et al., 2005; Mehta and Sibley, 2010; Haase et al., 2015; Baroni et al., 2018). In general, the functions of ADF/cofilins in actin regulation are depolymerization and severing of actin filaments (Bamburg, 1999). We have previously shown that *N. caninum* ADF (NcADF) has minor G-actin sequestering and more pronounced filamentous severing functions (Baroni et al., 2018) and these two functions were also observed, in major or minor degrees, in *Toxoplasma gondii* TgADF (Mehta and Sibley, 2010) and *Plasmodium falciparum* isoforms (PfADF1 and PfADF2) (Schüler et al., 2005; Singh et al., 2011; Wong et al., 2011; Wong et al., 2014). To strictly conduct their function in cells, ADF/cofilins are susceptible to the regulation of different mechanisms, comprising phosphorylation/dephosphorylation, binding to phospholipids (PIP_2_), pH variation, and oxidation/reduction (Kanellos and Frame, 2016; Bamburg et al., 2021). Point mutation at the serine 3 residue of TgADF, simulating the phosphorylation of this amino acid, inhibited the binding of the protein with actin, suggesting modulation of TgADF function by phosphorylation/dephosphorylation (Mehta and Sibley, 2010). On the other hand, this protein did not show interaction with PIP_2_ (Yadav et al., 2011). NcADF and TgADF affinities to F-actin were shown to be modulated by pH variations (Mehta and Sibley, 2010; Baroni et al., 2018). The role of oxidation/reduction (redox) in apicomplexan ADFs is unknown, although the role of redox conditions in human cofilin 1 has been established (Zdanov et al., 2010; Samstag et al., 2013; Bamburg et al., 2021). Upon oxidation, cofilin1 loses the ability of binding to actin and dimerizes. The dimerization is produced by an intermolecular disulfide bond between cysteines 39 and 147 (Pfannstiel et al., 2001) and the loss of the affinity with actin was attributed to methionine 115 (Luo et al., 2014) or cysteines 139 and 147 oxidation (Cameron et al., 2015). The sensitivity of cofilin1 to the redox conditions in the cell environment establishes this protein as a redox sensor, causing modifications in cellular functions which may vary between cell types (Samstag et al., 2013). As an oxidant, N-chlorotaurine (NCT) oxidizes cofilin and induces apoptosis in cells (Klamt et al., 2009; Luo et al., 2014).

NCT is a chlorinated amino acid derived from taurine, that is naturally produced by human leucocytes and has antiseptic properties (Gottardi and Nagl, 2010). Additionally, NCT has a limited spectrum of reactivity targeting most cysteine and methionine residues, aromatic amino acids, and amino groups of the proteins (Peskin and Winterbourn, 2001; Eitzinger et al., 2012; Lackner et al., 2022). In contrast, a similar mechanism induced by H_2_O_2_ usually damages proteins (Fligiel et al., 1984) and other biomolecules. Thus, NCT, which has already demonstrated protozoocidal activity against acanthamoebae, trichomonades and leishmaniae (Fürnkranz et al., 2008; Fürnkranz et al., 2009; Fürnkranz et al., 2011), is an interesting tool for the study of cysteine residues oxidation of proteins. As intracellular parasites, apicomplexans are exposed to potentially lethal levels of Reactive Oxygen Species (ROS), produced by the host innate defense cells (Shrestha et al., 2006; Mota et al., 2020). Nevertheless, the arsenal of the parasites’ antioxidant enzymes protects them against the oxidative burst produced by the host, being potential targets for treatment against intracellular parasites (Szewczyk-Golec et al., 2021; Venancio-Brochi et al., 2021). On the other hand, when the alterations in redox homeostasis are moderate, ROS might act as signaling molecules, participating in regulatory networks of cells (Winterbourn and Hampton, 2008). Even though signaling pathways have been investigated in mammalian cells (Kaludercic et al., 2014), the study of these processes in apicomplexan parasites is still emerging (Alves et al., 2021).

Here, we were interested in investigating whether recombinant NcADF is susceptible to oxidation using NCT or H_2_O_2_ as oxidizing agents. We showed that the protein was sensitive to redox variations forming reversible dimers and oligomers upon treatments with both oxidants. Additionally, both substances oxidized cysteine residues of NcADF to different degrees, but only NCT was able to significantly impair the binding of the recombinant protein to rabbit actin. The modulation of ADF function in apicomplexan parasites is poorly understood to date and its comprehension may potentially unveil cellular targets for the control of diseases caused by these parasites.

## Material and methods

### Chemicals

N-chlorotaurine (NCT) was obtained from two different sources. For dimerization assays, NCT was freshly prepared from the equimolar reaction between taurine (Sigma-Aldrich) and NaOCl (Sigma-Aldrich) in water (Klamt and Shacter, 2005). In the other assays, NCT was used as a crystalline sodium salt (MW 181.57 g/mol) (Gottardi and Nagl, 2002). Solutions of 250 mM NCT were freshly prepared in water and used for necessary dilutions in the experiments. H_2_O_2_ (30% v/v) was purchased from Sigma-Aldrich and fresh solutions of 500 mM were prepared in water before each experiment. All the other reagents were purchased from Sigma-Aldrich unless otherwise specified.

### Expression of recombinant NcADF

The recombinant actin-depolymerizing factor of *N. caninum* (NcADF) was expressed in a bacterial model as described, with minor modifications (Baroni et al., 2018). Briefly, *Escherichia coli* BL21(DE3) transformed with pET28(a)_NcADF recombinant plasmid was cultured in Luria Bertani (LB) medium and the protein expression was induced with 0.2 mM isopropyl β- d-1-thiogalactopyranoside (IPTG) at 18°C during 18 h. Bacteria were collected and sonicated (Branson Sonifier SLPe, Emerson Electric Co.) in lysis buffer (50 mM Tris, pH 7.0, 300 mM NaCl, 10% glycerol, 20 mM imidazole and Roche cOmplete mini protease inhibitor). The N-terminal 6X-his-tagged protein was purified by immobilized metal affinity chromatography (IMAC) (HisPur Ni-NTA Resin, Thermo-Fisher Scientific) and stored at -70°C in a storage buffer (20 mM Tris, pH 7.0, 30 mM NaCl, 5% glycerol; with or without 0.2 mM dithiothreitol - DTT) after quantification at 280 nm (ϵ_280nm_ = 13,980 M^-1^ cm^-1^) (Nanodrop 2000c, Thermo-Fisher Scientific).

### Oxidation of NcADF

The purified recombinant NcADF (25 µM) was oxidized with NCT or H_2_O_2_ as previously described (Eitzinger et al., 2012; Luo et al., 2014). NcADF was incubated with several concentrations of NCT or H_2_O_2_ for 30 min at room temperature. Subsequently, the solution was dried for 10 min at CentriVap Vacum Concentrator (Labconco) and the remaining protein was suspended in sample buffer (10% glycerol, 80 mM Tris, pH 6.8, 2% SDS, 0.01% bromophenol blue) with or without reductants. The solutions were incubated at 96°C for 3 min and resolved by SDS-PAGE. For NCT inactivation with sodium thiosulfate, a step was added to the protocol. After incubation with 10 mM NCT, the NcADF solutions were incubated for 10 min with 30 mM sodium thiosulfate.

### Protein analysis (western blotting)

NcADF was oxidized with 50 mM H_2_O_2_ (as described in 2.3) and 25 µM of the protein was resolved by SDS-PAGE without reductants. The gel content was transferred to the PVDF membrane (Immobilon 0.45 μM, Millipore) and NcADF was detected with anti-NcADF serum (1:15,000) (Baroni et al., 2018) followed by the incubation with anti-mouse IgG secondary antibody (anti-mouse IgG – whole molecule – peroxidase, an antibody produced in rabbit) (1:10,000). The transferred membranes were incubated with a chemiluminescent horseradish peroxidase (HRP) substrate (SuperSignal West Pico Chemiluminescent Substrate, Thermo-Fisher Scientific) and exposed to radiographic films.

### DTNB assay

The oxidation of NcADF cysteines was detected by the 5,5′-dithiobis-(2-nitrobenzoic acid) (DTNB or Ellman`s reagent) after incubation with NCT or H_2_O_2_. The recombinant NcADF (25 µM) was previously reduced with 2 mM tris(2-carboxyethyl)phosphine (TCEP) for 1 h at room temperature followed by desalinization in Sephadex G-25 (packed in PD-10, Cytiva Lifesciences) to remove TCEP from the solution. NcADF was eluted in 20 mM Tris pH 7.0 in 500 µl aliquots, which were tested with Bradford reagent (Bio-Rad) for the presence of the protein. To confirm when TCEP emerges from the desalinization column, a control solution without protein but containing TCEP was desalted in parallel and the presence of the reductant in the aliquots was tested with 2 mM DTNB. The aliquots with NcADF and without TCEP were stored at -70°C until its use. The reduced NcADF was incubated with NCT or H_2_O_2_ for 30 min and incubated, in duplicates, with 2 mM DTNB for 15 min in a 96-well plate (Non-treated surface; Thermo-Fisher Scientific). The reaction product of DTNB with the sulfhydryl groups of the cysteines was detected by absorbance at 512 nm (Biotek Epoch, Agilent) after 15 min incubation under light protection.

### Biotin-iodoacetamide (bio-IAM) assay

The oxidation of NcADF cysteines was evaluated by alkylation using biotin-iodoacetamide, as described (Hill et al., 2009). NcADF was reduced by TCEP and then desalinized (see DTNB assay). Subsequently, the recombinant protein was oxidized with NCT or H_2_O_2_ for 30 min at room temperature. The excess of NCT and H_2_O_2_ were inactivated with 12 mM histidine/methionine and 2 U catalase, respectively, for 20 min. After inactivation, the protein solutions were incubated with 0.5 µM biotin-iodoacetamide (bio-IAM) for 20 min at room temperature. Bio-IAM was quenched by the addition of 30 mM β-mercaptoethanol (Gibco Thermo-Fisher Scientific). Afterward, the reactions were dried in the vacuum concentrator and the contents were suspended in a non-reductant sample buffer at a final concentration of 25 µM NcADF. The samples were resolved by SDS-PAGE and transferred to PVDF membranes, which were stained with DB-71 (0,008% w/v Direct Blue 71, 10% acetic acid, 40% ethanol). The alkylation of cysteines was detected by streptavidin-peroxidase (1:20,000; Thermo-Fisher Scientific) and observed after luminol incubation in radiographic films.

### Preparation of rabbit muscle actin

The rabbit actin from skeletal muscle was isolated from acetone powder as previously described by (Pardee and Spudich, 1982). The “acetone powder” was produced using the skeletal muscles from a rabbit acquired in a local butchery. Briefly, the powder was suspended in G buffer (2 mM Tris, pH 8.0, 0.5 mM ATP, 0.2 mM CaCl_2_, 0.5 mM DTT) and actin was isolated after induction of polymerization with 50 mM KCl. Then, polymerized actin was separated from tropomyosin in 800 mM KCl. The filaments of actin were sedimented by ultracentrifugation at 100,000 *g* (TL-100 Ultracentrifuge; Beckman Coulter Inc.; rotor 70.1 TI) at 4°C for 1 h. The sediment containing actin filaments was suspended in G buffer and dialyzed against the same buffer at 4°C for 48 h for actin depolymerization. Afterward, the solution was again ultracentrifuged at 100,000 *g* for 1 h and the actin in the supernatant was concentrated, quantified (ϵ_290nm_ = 26.600 *M^-l^* cm^-1^), and stored at -70°C in small aliquots.

### Microplate protein-binding assay

The previously reduced recombinant NcADF was oxidized with NCT or H_2_O_2_ (description in 2.3) in G buffer (without DTT) and the excess of the oxidants was inactivated with histidine/methionine solution or catalase, respectively. The reduced NcADF control was obtained after incubation of the protein with G buffer containing DTT. Afterward, the oxidized and reduced protein was serially diluted (20-0.312 µg/ml). NcADF was used in the microplate protein-binding assay, which was performed as described before, with modifications (Biesiadecki and Jin, 2011). Briefly, 100 µl of 2.5 µg/ml monomeric actin (G-actin in G buffer) was immobilized in a 96-well plate (NUNC MaxiSorp flat-bottom, Thermo-Fisher Scientific) at 4°C for 18 h. The wells were washed once with 200 µl G buffer-T (G buffer with 0.1% Tween-20) and blocked overnight at 4°C with 150 µl 3% BSA in G buffer-T. After the blocking, the wells were washed three times with G buffer-T followed by the addition of NcADF dilutions, in duplicate, and incubated at 4°C for 18 h. After three washes, the wells were incubated with anti-NcADF serum (1:1,000, in G buffer-T with 0.1% BSA) for 1 h at room temperature, followed by incubation with anti-mouse secondary antibody (1:10,000) for 45 min. The colorimetric signal was produced after the addition of TMB (OptEIA TMB Substrate Reagent Set, BD Biosciences) and quantified at 650 nm after 30 min incubation. The results were analyzed using the nonlinear fit log vs. response procedure in Prism Software (GraphPad). The statistical analysis was performed using One Way ANOVA.

### Protein cross-linking

For this assay, 1-ethyl-3-[3-dimethylaminopropyl]carbodiimide hydrochloride (EDC) was used as a zero-length crosslinker. NcADF was oxidized with NCT or H_2_O_2_ in G buffer and the excess oxidants were inactivated as previously described in this study. The oxidized or reduced NcADF was incubated with equimolar G-actin for 18 h at 10°C and 350 rpm (Eppendorf ThermoMixer). After the reaction, 1 mM EDC was added to the protein mixture and incubated for 15 min at 37°C. The addition and incubation with EDC were repeated one more time. Subsequently, 9 mM glycine was added and the reactions were resolved by SDS-PAGE. The gels were stained with Coomassie G-250.

### PI-actin polymerization assay

The polymerization assay was performed as described in Baroni et al. (2018), with modifications. In a 96-well black plate, 15% PI-actin (Pyrene-labeled actin from rabbit muscle, BioVision) was added (final concentration of 10 µM) to an equimolar concentration of NcADF previously oxidized with NCT. The mixture of proteins was incubated for 10 min at room temperature. Afterward, 10X ME (500 mM MgCl_2_, 2 mM EGTA) was added to the proteins. After a 2-min incubation, the actin polymerization was induced with 10X KMEI (500 mM KCl, 10 mM MgCl_2_, 10 mM EGTA, 100 mM imidazole, pH 7.0). The fluorescence (ex/em 365/407 nm) was detected in a microplate reader (Spectramax i3, Molecular Devices LLC).

### Computational analysis

The global alignment of NcADF (ToxoDB ID NCLIV_012510/GenBank ID XP_003881486) and human cofilin 1 (GeneBank ID 1072) amino acid sequences was performed using MegAlign 7.1.0 (Lasergene, DNASTAR) and visualized in ESPript (Robert and Gouet, 2014). The software Pymol 1.1r.1 (Molecular Graphics System, Schrödinger, LLC) was employed to align and visualize the tertiary structures of human cofilin 1 (PDB: 1q8x) and NcADF (Baroni et al., 2018).

## Results

### Recombinant NcADF forms dimers when oxidized

**Figure 1:**
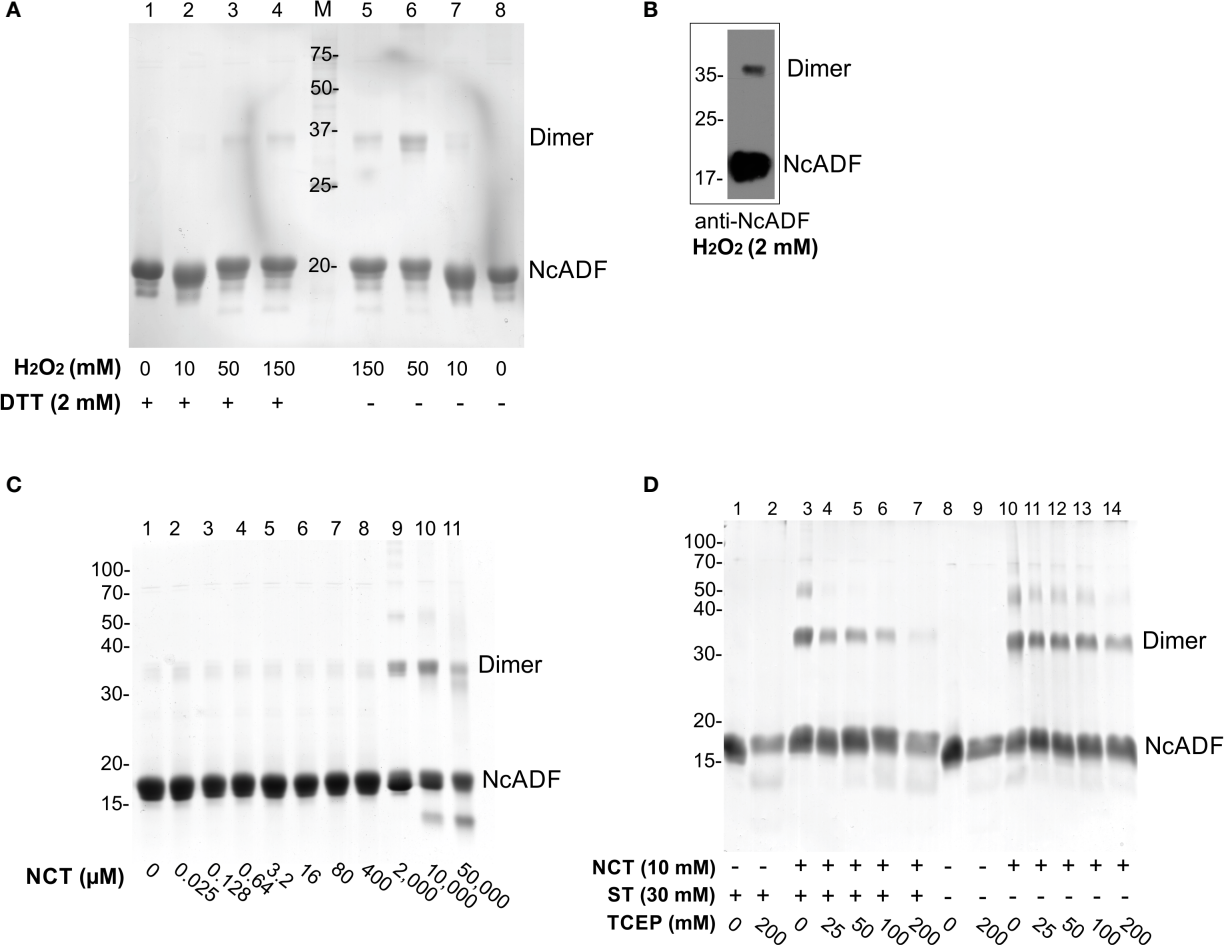
Dimerization of oxidized recombinant NcADF. NcADF produced and stored without reductant was incubated with oxidants. **(A)** NcADF was incubated with 0-150 mM of H_2_O_2_ for 30 min. **(B)** NcADF was incubated with 2 mM H_2_O_2_ and NcADF was detected by western blotting using NcADF antiserum (1:15,000) and anti-mouse secondary antibody conjugated to HRP (1:10,000). **(C)** NcADF was incubated with 0-50 mM NCT for 30 min. **(D)** NcADF was incubated with 10 mM NCT, followed by NCT inactivation with sodium thiosulfate (ST) and reduction with TCEP. 15% SDS-PAGE stained with Coomassie G-250 **(A, C, D)**.

To evaluate the susceptibility of the recombinant NcADF to oxidation, the protein was treated with N-chlorotaurine (NCT) or H_2_O_2_ and observed by SDS-PAGE. NcADF was expressed as an 18-kDa protein (Baroni et al., 2018) and the treatment with a large range of H_2_O_2_ concentrations induced the formation of a band with 36 kDa (**Figure 1A**, lanes 5-8), indicating that, under oxidation, NcADF forms dimers. The treatment with 150 mM H_2_O_2_ produced a less intense dimer band compared to 50 mM of the oxidant (**Figure 1A**, lanes 5 and 6), indicating that higher concentrations of H_2_O_2_ may be altering the protein activity and producing an excess of oxidation. The detection of this protein by the NcADF antiserum in both bands confirmed the dimerization (**Figure 1B**). To assess if the dimer formation was reversible, the oxidized NcADF was, subsequently, reduced with dithiothreitol (DTT) (**Figure 1A**, lanes 1-4). In this condition, the intensity of the 36-kDa bands was reduced when compared to the counterparts without DTT, confirming the reversibility of the dimerization.

NCT was previously shown to oxidize human cofilin1 (Klamt et al., 2009). In order to evaluate the NcADF dimerization by NCT, several concentrations of this substance were tested in recombinant NcADF produced without DTT in the storage buffer. Under non-reduced conditions, a discrete dimer band was formed (**Figure 1C**, lane 1). The use of lower concentrations of NCT did not alter the formation of dimers, but concentrations above 2 mM induced more intense bands of 36 kDa (**Figure 1C**, lanes 9-11). To assess whether the formation of dimers by NCT was also reversible, as observed with H_2_O_2_, the oxidized NcADF was reduced by tris(2-carboxyethyl)phosphine (TCEP), which disrupts the disulfide bonds, after the oxidation with NCT. In parallel, the excess of NCT in the solution was inactivated by sodium thiosulfate (ST) (Böttcher et al., 2017). With NCT inactivation, the presence of a higher concentration of TCEP significantly reduced the intensity of the dimer and oligomers bands (**Figure 1D**, lane 7) compared to the oxidation control (**Figure 1D**, lane 3). Moreover, the decrease in band intensity was less intense in ST-free samples (**Figure 1D**, lanes 11-14) compared to the ST supplemented ones (**Figure 1D**, lanes 4-7).

### NcADF cysteine residues are oxidized

**Figure 2:**
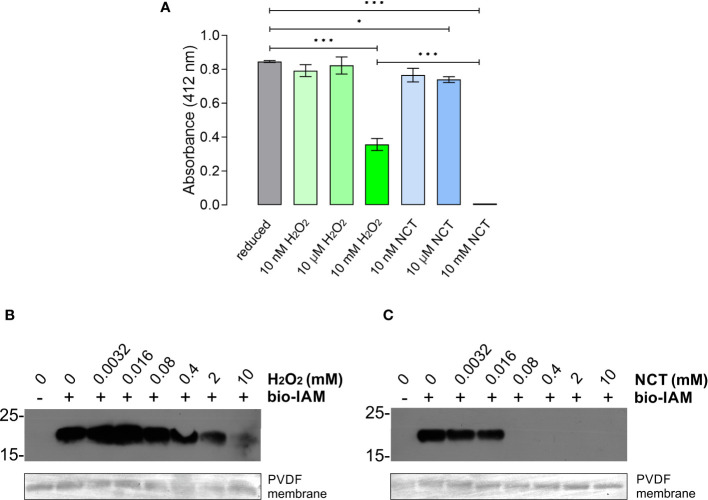
Oxidation of NcADF cysteine residues. Recombinant NcADF was reduced with TCEP. After removing TCEP, the protein was oxidized with NCT or H_2_O_2_. **(A)** NcADF was incubated with NCT or H_2_O_2_ and the cysteine residues´ oxidation was detected by three independent DTNB assays performed in duplicate. The bars represent the mean ± SD. The *** indicates p ≤ 0.0001 and the * indicates p ≤ 0.05 in Tukey’s test between the treatments. **(B, C)** NcADF was incubated with 0-10 mM H_2_O_2_ **(B)** or NCT and 25 µM NcADF in 20 µl non-reductant sample buffer was loaded in the gel **(C)**. The oxidants were inactivated with catalase or histidine/methionine, respectively, and alkylated with 0,5 µM bio-IAM. The protein was run in 15% SDS-PAGE and transferred to PVDF membranes. The alkylation was detected by streptavidin-HRP (1:20,000). For protein level control, the PVDF membranes were stained with Direct Blue 71.

DNTB was used to investigate whether NCT and H_2_O_2_ treatments would oxidize cysteine residues of NcADF. This substance reacts with cysteine’s free sulfhydryl groups to yield the yellow compound 2-nitro-5-thiobenzoic acid (TNB), detected by colorimetry. The oxidized cysteine does not react with DTNB, impairing or reducing the absorbance. Nanomolar concentrations of NCT or H_2_O_2_ had no significant effect on NcADF thiol oxidation, whereas micromolar concentration of NCT, but not of H_2_O_2_, was sufficient to oxidize the protein (p ≤ 0.05) (**Figure 2A**). When the protein was treated with 10 mM NCT or H_2_O_2_, the absorbance was significatively (p ≤ 0.0001) reduced in both treatments compared to the reduced protein (**Figure 2A**). Nevertheless, at the same concentration, the treatment with NCT had a more pronounced effect (p ≤ 0.0001) compared to the treatment with H_2_O_2_ (**Figure 2A**). To confirm these observations, the levels of cysteine oxidation were also monitored by alkylation with bio-IAM. The decrease of the NcADF band intensity indicates thiol groups´ oxidation of the cysteine residues. The treatment with 2 and 10 mM H_2_O_2_ reduced the intensity of the bands (**Figure 2B**). Likewise, the NCT treatment completely abrogated the signal with concentrations above 0.08 mM (**Figure 2C**). The results using alkylation confirmed the ones obtained with DTNB. NCT and H_2_O_2_ oxidized NcADF cysteine residues, but NCT demonstrated a higher oxidation potential.

### NcADF oxidation impairs its binding with actin

**Figure 3:**
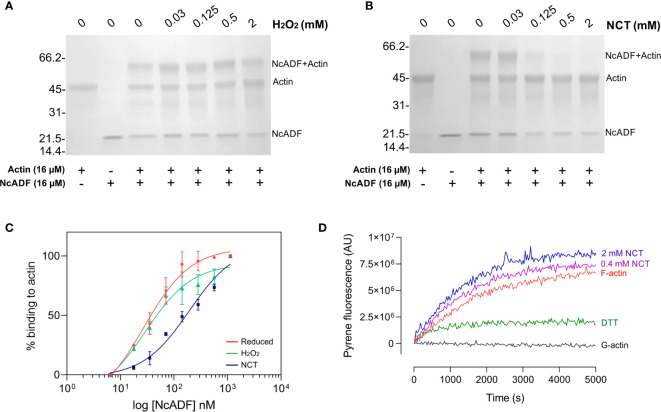
Binding and dynamic of oxidized NcADF and actin. Previously reduced NcADF was incubated with H_2_O_2_ or NCT and the binding with actin was assessed. **(A, B)** NcADF (16 µM) was treated with 0-2 mM H_2_O_2_ **(A)** or NCT **(B)**. After inactivation with catalase **(A)** or histidine/methionine **(B)**, NcADF was incubated with 16 µM G-actin for 18 h EDC (2 mM) was added to the reaction, which was resolved by 15% SDS-PAGE and stained with Coomassie G-250. **(C)** G-actin (2.5 µg/ml) was immobilized and incubated with 0-20 µg/ml NcADF oxidized with either 2 mM NCT or H_2_O_2_. NcADF was detected with NcADF antiserum (1:1,000) and anti-mouse secondary antibody-HRP (1:10,000). Reduced NcADF EC50 (red circles) = 27.6 nM; NCT treated NcADF EC50 (blue squares) = 192.4 nM; H_2_O_2_ treated NcADF (green triangles) = 29.5 nM. Results from two (2) experimental replicates. **(D)** PI-G-actin (15% PI-actin; 10 µM) was mixed with 10 µM NcADF previously treated with 0-2 mM NCT. The actin polymerization was induced and the fluorescence was measured over time. Grey line = G-actin control without NcADF; green line = actin polymerization with reduced NcADF; red line = actin polymerization without NcADF; purple line = actin polymerization with NcADF (oxidized with 0.4 mM NCT); blue line = actin polymerization with NcADF (oxidized with 2 mM NCT).

The ability of NCT- and H_2_O_2_-treated NcADF to bind to rabbit G-actin was determined using the crosslinker EDC. The binding of NcADF (18 kDa) and actin (42 kDa) produced a band containing the sum of the molecular weight of both proteins (60 kDa). Moreover, binding was proportional to the intensity of this band. The treatment of NcADF with up to 2 mM H_2_O_2_ did not impair the formation of the 60-kDa band (**Figure 3A**). However, the same concentration of NCT reduced the band intensity in a concentration-dependent manner (**Figure 3B**). In this experiment, the dimer formation was not as clear as in **Figure 1**, since the mass of protein applied was lower. The binding of these proteins was also investigated in non-equilibrium conditions. NCT presented higher EC50 (half-maximum effective concentration) compared to H_2_O_2_ (192.4 nM and 29.53 nM, respectively). In comparison, the EC50 for reduced NcADF was 27.65 nM (**Figure 3C**). These data suggest that the ability of NcADF to bind to G-actin decreases under the oxidation of that protein. To evaluate the role of oxidized NcADF in actin dynamics, the recombinant protein was oxidized or not and applied in the polymerization assay. The reduced NcADF decreased the actin fluorescence, while the oxidized NcADF accelerated the initial rate of polymerization (**Figure 3D**). This alteration in actin polymerization by reduced or oxidized NcADF suggests that the oxidized protein did not bind to actin. Furthermore, the effect of the protein in actin polymerization was produced by the remaining reduced active NcADF.

### Cysteines are positioned differently in NcADF and human cofilin 1

**Figure 4:**
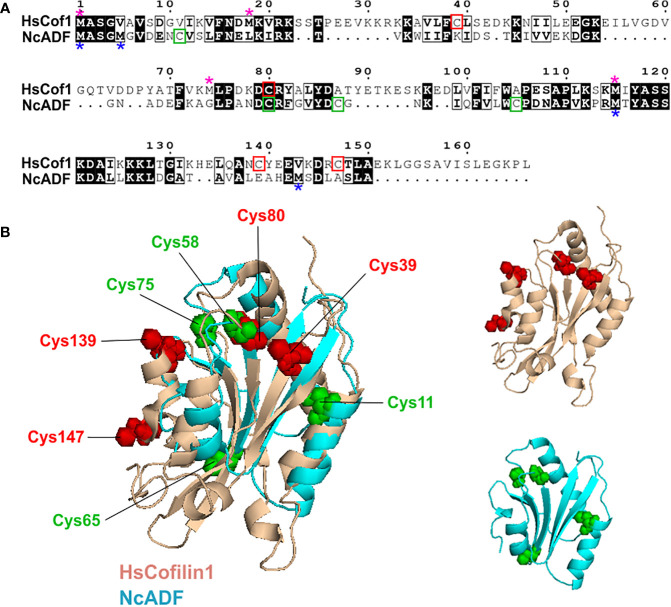
Comparison of cysteines’ position between NcADF and human cofilin 1. **(A)** Alignment of human cofilin 1 (HsCof1) and NcADF primary sequences. The HsCof1 cysteines (red rectangles) and methionines (pink asterisks), as well as NcADF cysteines (green rectangles) and methionines (blue asterisks) are highlighted. **(B)** The cartoon representations of NcADF (Baroni et al., 2018) and HsCofilin1 (PDB: 1q8x) tertiary structures were aligned. The two structures are shown independently on the right. The cysteines are represented as green spheres (NcADF) or red spheres (HsCofilin1) and their relative positions are described in the image.

The oxidation of human cofilin 1 is a known mechanism of protein activity regulation (Samstag et al., 2013; Bamburg et al., 2021). However, considering that NcADF and mammalian cofilin share less than 30% identity (Baroni et al., 2018) we investigated whether the position of cysteine residues in the structures of the two proteins is conserved. The alignment of the primary sequences revealed that only the Cys 58 (Cys 80 in cofilin 1) is conserved (**Figure 4A**). To assess whether the position of cysteine residues is maintained between the tertiary structures, the structure of both proteins was aligned and confirmed that Cys 58 (NcADF) and Cys 80 (cofilin 1) occupy correspondent positions in the molecules (**Figure 4B**). The additional cysteines occupy distinct portions of the molecules, with emphasis on cofilin Cys 139 and 147, absent in NcADF, which were identified as responsible, when oxidized, to reduce the binding of the protein with actin (Cameron et al., 2015).

## Discussion

In this study, we showed that the *Neospora caninum* actin-depolymerization factor (NcADF) is modulated by oxidation and reduction (redox) conditions. ADF/cofilins are proteins primarily related to the regulation of actin dynamics in cells, performing a decisive function in actin depolymerization or severing (Maciver and Hussey, 2002; Kanellos and Frame, 2016). In parallel with the function of actin regulators, these proteins require themselves a regulatory context to strictly conduct their role in cell processes. Phosphorylation/dephosphorylation, variation of pH and binding to phospholipids were, at a certain point, investigated in ADF of apicomplexan parasites, including *N. caninum* (Mehta and Sibley, 2010; Yadav et al., 2011; Baroni et al., 2018). However, modulation by reduction and oxidation was an unknown condition of apicomplexan ADFs so far. Oxidative modifications of redox-sensitive proteins are chemical mechanisms that can mediate cell processes, including cell signaling (Schieber and Chandel, 2014). These proteins are also susceptible to oxidative post-translational modifications typically produced by reactive oxygen species (ROS). In proteins, several amino acids are susceptible to oxidation including methionine, histidine, tryptophan, phenylalanine, and cysteine. Remarkably, cysteine residues are candidates for reversible modifications in proteins. Redox reactions of cysteines are important mechanisms of regulation implicated in protein function modulation and signaling pathways in cells (Fra et al., 2017). Here, to evaluate the susceptibility of NcADF to oxidation, two oxidants were used: H_2_O_2_ and N-chlorotaurine (NCT). The protein aggregation in homodimers and oligomers under oxidative conditions may be caused by the formation of intermolecular disulfide bonds. The addition of the reductants DTT or tris(2-carboxyethyl)phosphine (TCEP), both disulfide bridge-breaking agents, reversed the dimerization, indicating that it was formed by disulfide bonds. Also, the experiment was performed using NcADF stored in a buffer without reductants and the protein formed dimers without oxidation treatment suggesting a high sensitivity to the redox environment. The formation of intramolecular disulfide bonds was demonstrated in human cofilin 1 (Klemke et al., 2008; Klamt et al., 2009) and may also be formed in NcADF. The change in migration velocity of NcADF in the gel after the treatment with higher concentrations of oxidants compared to the reduced control is indicative of intramolecular bond formation. Additionally, the NcADF residues likely to form an intramolecular disulfide bond are Cys 58 and 75, which are positioned in different portions of the molecule when compared to the cofilin 1 residues (Cys 39 and 80) in similar conditions (Bernstein et al., 2012).

NcADF contains four cysteine residues at positions 11, 58, 65 and 75, being the last three ones exposed in the folded structure as potential targets for oxidation (**Supplementary Material**). We investigated whether NcADF cysteine residues were prone to oxidation and we found that they are oxidized upon NCT and higher concentration of H_2_O_2_. However, interestingly, DTNB and bio-IAM methods showed that NCT was more efficient in producing cysteine oxidation compared to the same concentrations of H_2_O_2_. This difference can be explained by the specificity of NCT in oxidizing cysteine and methionine residues and chlorinating tyrosine, phenylalanine, histidine, and tryptophan ones and amino groups (Peskin and Winterbourn, 2001; Eitzinger et al., 2012; Lackner et al., 2022), while H_2_O_2_ has a broad and unspecific effect (Fligiel et al., 1984). Cofilin 1 cysteine residues are susceptible to oxidation by NCT and H_2_O_2_ (Klemke et al., 2008; Klamt et al., 2009; Luo et al., 2014; Cameron et al., 2015). Using cofilin 1 pre-exposed to H_2_O_2_, Cameron et al. (2015) demonstrated, with the bio-IAM method, that cysteines were not completely oxidized upon a similar range of treatment, in accordance with the results presented here. In addition, in the *Plasmodium falciparum* ADF1 (PfADF1) crystal structure analysis, cysteine residues 24 and 34 were found to be oxidized, even though these residues are not conserved between PfADF1 and NcADF sequences (Singh et al., 2011; Baroni et al., 2018).

We showed before that NcADF binds weakly to rabbit G-actin and accelerates the initial rate of PI-actin polymerization (Baroni et al., 2018). The affinity of human cofilin 1 with actin is regulated by the oxidation of cysteine and methionine residues (Klemke et al., 2008; Klamt et al., 2009; Luo et al., 2014; Cameron et al., 2015). In this study, we investigated whether the affinity of NcADF to rabbit G-actin was altered by the pre-treatment of the first protein with the oxidant substances. The rabbit actin was employed due to its established protocols for the study of ADF/cofilins (Maciver et al., 1998; Ressad et al., 1998; Yamashiro et al., 2005; Tammana et al., 2008; Dai et al., 2013), including the apicomplexan orthologs (Schüler et al., 2005; Mehta and Sibley, 2010; Wong et al., 2011). The oxidation of NcADF cysteine residue by NCT affects the ability of this protein to interact to actin, suggesting that oxidation can be a regulator mechanism of NcADF activity. In our previous study, we showed a weak affinity between NcADF and rabbit actin using a different method (Baroni et al., 2018). In the present study, we used a previously reduced NcADF in the control reactions and applied a longer incubation time to the protein mixture, improving the affinity of these two proteins. We also assessed the effect of oxidized NcADF on pyrene actin (PI-actin) formation of filaments and observed that the reduced NcADF decreased the initial rate of actin polymerization. The inhibition of this process is probably caused by monomer sequestering, in accordance with the binding results. Alternatively, the decrease in the fluorescence compared to the actin control may be caused by the interaction of NcADF and PI-actin filaments, quenching the fluorescence signal (Carlier et al., 1997; Blanchoin and Pollard, 1999). The treatment of NcADF with NCT increased the polymerization rate of actin, probably caused by a weaker affinity of the oxidized protein to actin monomer and by the severing of filaments. This data supports evidence that the oxidation of NcADF cysteine residues can modulate the activity of the protein in actin dynamics. This data also diverged from the results we obtained in our previous study (Baroni et al., 2018). At similar concentrations, NcADF was shown to improve the initial rate of polymerization and analogous results were observed in TgADF and PfADF2 (Mehta and Sibley, 2010; Singh et al., 2011). However, the data from our previous and actual studies cannot be directly compared due to significant methodology divergences. In general, it highlights the importance of examining the redox status of ADF/cofilins before the investigation to guarantee no bias caused by involuntary amino acids oxidation.

Our results reveal that, differently as previously inferred (Cameron et al., 2015), the modulation by oxidation may not be restricted to cofilin and higher species. Although the position of the cysteine residues in ADF/cofilins is not fully conserved between species and isoforms (Maciver and Hussey, 2002), the modulation of these proteins by oxidation may be a condition maintained along with the evolution. In addition, apicomplexan ADFs have many aspects that diverge from the function of metazoan ADF/cofilins (Kumpula and Kursula, 2015). This indicates that the discrepancy in cysteine residues configuration can determine different properties of the protein modulation under redox conditions, which is a key feature to be observed in potential drug targets. Furthermore, apicomplexan parasites use the actin-myosin motor to invade host cells and are challenged by the oxidative intracellular cell environment as well as by the ROS produced by the host’s innate immune system (Bosch et al., 2015). The impact of the environmental redox in apicomplexan cell cycle has been investigated, albeit it is still incompletely understood. Recently, an interplay between extracellular redox and intracellular Ca^+2^ signaling was demonstrated (Alves et al., 2021). Our findings showed that a regulator component of actin dynamics can be modulated by oxidation, indicating a potential connection between redox environment and actin activity regulation.

## Data availability statement

The original contributions presented in the study are included in the article/**Supplementary Material**. Further inquiries can be directed to the corresponding author.

## Author contributions

LB and AY contributed to the conceiving of the experiments. LB designed the experiments and performed data analysis. LB and PGA-F performed the experiments. LP and MN contributed with methods and reagents. LB wrote the manuscript. All authors contributed to the manuscript’s critical revision, read, and approved the submitted version.

## Funding

This research was supported by public funding from Sao Paulo Research Foundation (FAPESP) (Grant processes number: 2018/21020-6 and 2022/12746-6, Fundação de Amparo à Pesquisa do Estado de São Paulo).

## Acknowledgments

We would like to acknowledge Maraísa Palhão Verri for the technical assistance. We are also thankful for the Dr. Fabiola Attié de Castro (School of Pharmaceuticals Sciences of Ribeirao Preto) for the accession to the Spectramax i3.

## Conflict of interest

The authors declare that the research was conducted in the absence of any commercial or financial relationships that could be construed as a potential conflict of interest.

## Publisher’s note

All claims expressed in this article are solely those of the authors and do not necessarily represent those of their affiliated organizations, or those of the publisher, the editors and the reviewers. Any product that may be evaluated in this article, or claim that may be made by its manufacturer, is not guaranteed or endorsed by the publisher.
