# Supplementary material for: Recombinant actin-depolymerizing factor of the apicomplexan Neospora caninum (NcADF) is susceptible to oxidation
Source: Front Cell Infect Microbiol. 2022 Dec 19;12:952720. doi: 10.3389/fcimb.2022.952720 (PMC9806845; doi:10.3389/fcimb.2022.952720)
Supplement: Supplementary file 1 [file Image_1.pdf]

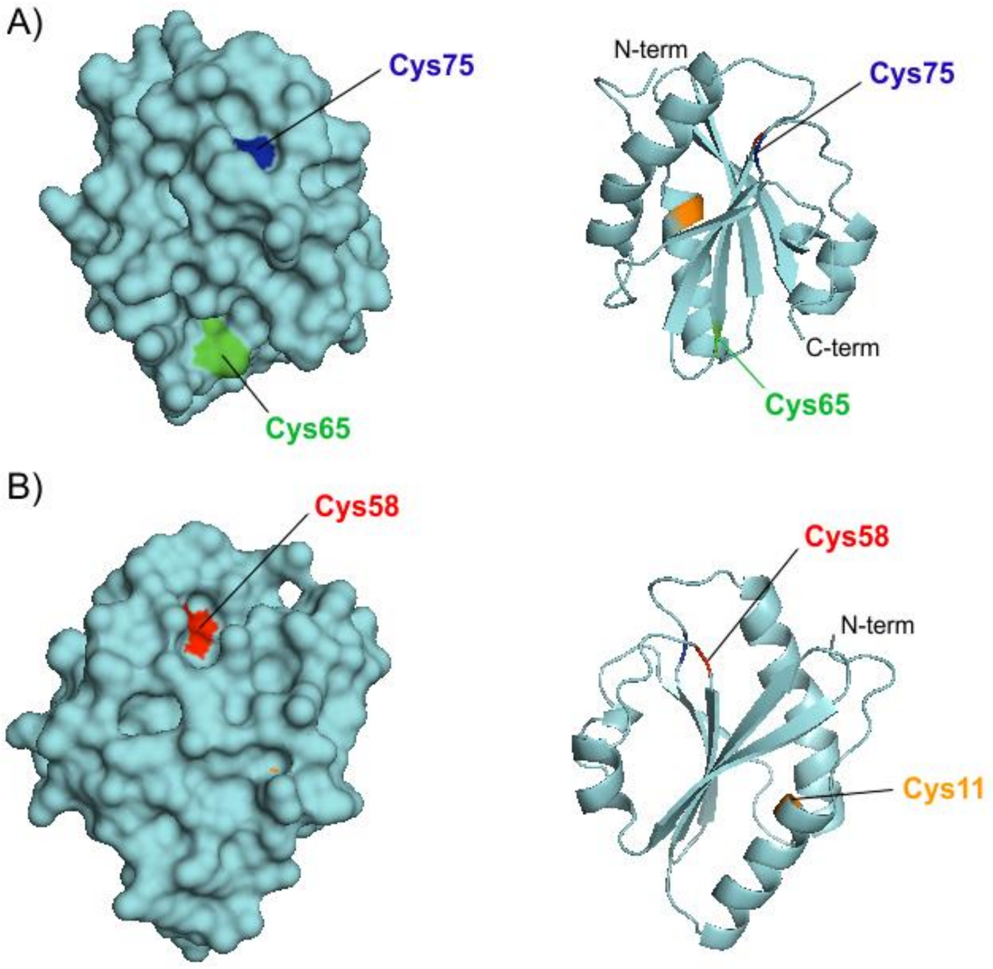


**Supplementary figure 1.** Cysteines' position in the NcADF tertiary structure. The structure of NcADF was generated by homology modeling (Baroni et al., 2018) and visualized in Pymol (Schrödinger and DeLano, 2020). **A)** Cys 65 (green) and 75 (blue) are highlighted in the surface and cartoon representations of NcADF. **B)** Cys 58 (red) and 11 (orange) are shown in the surface and/or cartoon representations of NcADF.

## References

Baroni, L., Pereira, L. M., Maciver, S. K., and Yatsuda, A. P. (2018). Functional characterisation of the actin-depolymerising factor from the apicomplexan Neospora caninum (NcADF). *Molecular and Biochemical Parasitology* 224, 26–36. doi: 10.1016/j.molbiopara.2018.07.008.

Schrödinger L, DeLano W. PyMOL [Internet]. 2020. Available from: http://www.pymol.org/pymol
